# Tidal swings of endotracheal tube cuff pressure during pressure support ventilation: A CARE-compliant case report

**DOI:** 10.1097/MD.0000000000047051

**Published:** 2026-01-09

**Authors:** Rui-Zhi Zhang, Shan-Shan Xu, Ming-Yue Miao, Hai-Yang Liu, Yun-Long Yue, Jian-Xin Zhou

**Affiliations:** a Emergency and Critical Care Center, Clinical and Research Center on Acute Lung Injury, Beijing Shijitan Hospital, Capital Medical University, Beijing, China; b Department of Radiology, Beijing Shijitan Hospital, Capital Medical University, Beijing, China.

**Keywords:** endotracheal tube cuff pressure, esophageal pressure, inspiratory effort, monitoring, pressure support ventilation

## Abstract

**Rationale:**

During assisted mechanical ventilation, such as pressure support ventilation (PSV), it is crucial to align the ventilator support with the patient’s inspiratory effort. Therefore, continuous monitoring of effort at the bedside is essential.

**Patient concerns:**

In this report, we present a 43-year-old woman undergoing pressure support ventilation via an oral endotracheal intubation (7.5-mm I.D., depth of 22 cm from central incisors, and cuff inflated to a pressure of 30 cmH_2_O).

**Diagnoses:**

Acute hypoxemic respiratory failure secondary to pneumonia.

**Interventions:**

Pressure support ventilation was initiated to evaluate weaning readiness, and esophageal pressure (P_ES_) monitoring was established to assess inspiratory effort. Endotracheal tube cuff pressure (P_CUFF_) was monitored. During the inspiratory phase of pressure support ventilation, negative tidal swings were noticed in the P_CUFF_ tracing, whose shapes were consistent with those of P_ES_. A downward pressure support titration was performed from 15 cmH_2_O to 5 cmH_2_O in 2 cmH_2_O increments. P_ES_ and P_CUFF_ were simultaneously recorded.

**Outcomes:**

As the pressure support decreased, the negative tidal swing of P_ES_ increased markedly. At pressure support of 13 cmH_2_O and 15 cmH_2_O, the tidal swing of P_CUFF_ exhibited a plain or slightly positive shape. However, as the pressure support further decreased to levels below 11 cmH_2_O, the tidal swing of P_CUFF_ changed to a negative shape, and the magnitude increased markedly. The tidal swings of P_CUFF_ and P_ES_ were closely correlated across different pressure support levels. Chest radiography confirmed that the endotracheal tube cuff position was below the thoracic inlet and within the thoracic cage.

**Lessons:**

This case illustrates the potential application of P_CUFF_ in assessing inspiratory effort during pressure support ventilation. However, due to the preliminary nature of this single observation, further study in a large cohort is warranted.

## 1. Introduction

As a primary mode of assisted mechanical ventilation, pressure support ventilation (PSV) is widely used in the intensive care unit.^[1]^ The successful implementation of PSV depends on matching the provided ventilator support with the patient’s inspiratory effort. Studies have shown that both excessive and insufficient inspiratory effort could result in ventilator-induced lung and diaphragm injury, underscoring that monitoring inspiratory effort is essential and may facilitate lung and diaphragmatic protection.^[2]^ Currently, esophageal pressure (P_ES_)-derived metrics represent the gold standard for assessing inspiratory effort in clinical studies.^[3]^ However, P_ES_ monitoring requires specialized equipment and involves complex procedures, making it unsuitable for routine clinical use.

Several airway pressure (P_AW_)-based parameters for monitoring inspiratory effort, such as the negative P_AW_ generated during the first 100 milliseconds and the maximal negative swing of P_AW_ against an end-expiratory airway occlusion, have been introduced in recent years.^[4,5]^ However, these measurements require airway occlusion maneuvers and can only be obtained intermittently. There is a clinical need for a convenient and continuous monitoring method to assess inspiratory effort at the bedside.

## 2. Case report

This case report was compliant with the CARE (CAse REport) Statement.^[6]^ Written informed consent was obtained from the patient.

A 43-year-old woman with acute hypoxemic respiratory failure secondary to pneumonia was mechanically ventilated via an oral endotracheal tube (Mallinckrodt: LOT 15C0090JZX, COVIDIEN LLC., Shanghai, China, 7.5-mm I.D., depth of 22 cm from central incisors, and cuff inflated to a pressure of 30 cmH_2_O). PSV was initiated to evaluate weaning readiness with pressure support of 10 cmH_2_O, positive end-expiratory pressure of 5 cmH_2_O, flow-trigger of 2 L/min, inspiration-to-expiration cycle-off at 25% of the peak inspiratory flow, and fraction of inspired oxygen of 0.4. P_ES_ monitoring was established to assess inspiratory effort using a standardized technique with an esophageal balloon catheter (Cooper catheter: LOT 177405, Cooper Surgical, Trumbull).^[3]^ P_ES_, P_AW_, and airflow signals were synchronously recorded using a heated Fleisch pneumotachograph (Vitalograph Inc, Lenexa) and 2 pressure transducers (KleisTEK Engineering, Bari, Italy) positioned at the distal end of the endotracheal tube. We also recorded endotracheal tube cuff pressure (P_CUFF_) with an additional pressure transducer connecting to the cuff. Signals were displayed continuously and saved on a laptop for offline analysis, at a sample rate of 200 Hz (ICU-Lab 2.5 Software Package, KleisTEK Engineering, Bari, Italy).

**Figure 1.**
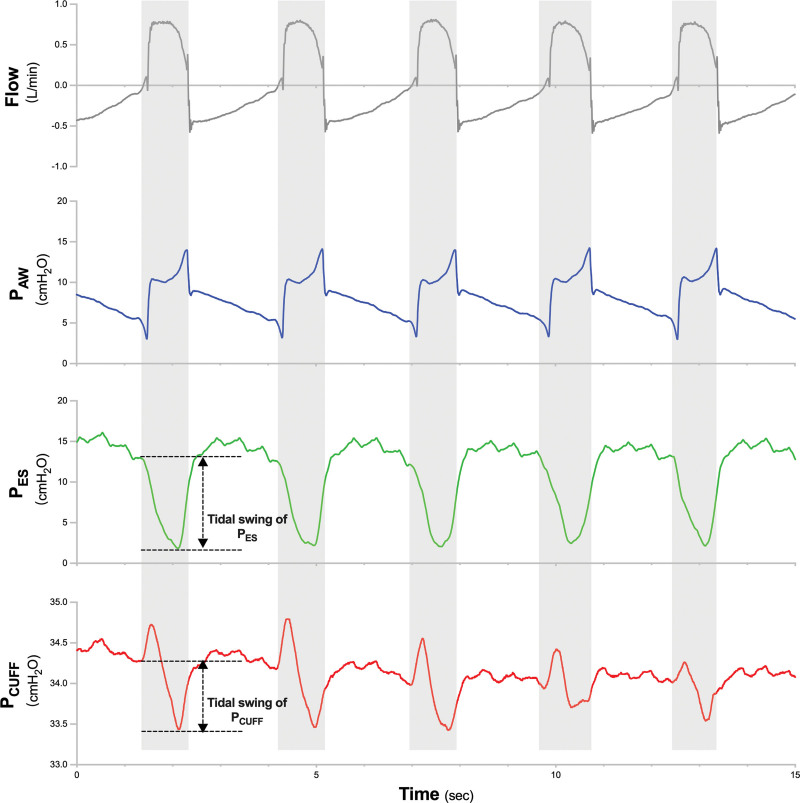
Flow, P_AW_, P_ES_, and P_CUFF_ during pressure support ventilation. The gray zones represent the inspiratory phase of mechanical ventilation. The onset of inspiratory effort is defined as the point of negative deflection of P_ES_ with a rapid change in slope.^[3,4]^ The tidal swings of P_ES_ and P_CUFF_ were labeled. P_AW_ = airway pressure, P_CUFF_ = endotracheal tube cuff pressure, P_ES_ = esophageal pressure, PSV = pressure support ventilation.

During the inspiratory phase under PSV, negative tidal swings were noticed in P_CUFF_ tracing, whose shapes were consistent with those of P_ES_ (Fig. 1).

According to our local clinical routine, a pressure support titration was performed to assess the patient’s inspiratory effort in response to ventilator support.^[4]^ Before the titration, the endotracheal cuff was manually checked using a Cuff Pressure Gauge Pocket (VBM Medizintcechnik GmbH, German, LOT 0000245739), and the cuff was inflated to a pressure of 30 cmH_2_O. Then the cuff was connected to the pressure transducer.

With other ventilator settings remaining unchanged, the pressure support was adjusted downward from 15 cmH_2_O to 5 cmH_2_O in 2 cmH_2_O increments with a 3-minute stabilization period at each level. We measured the tidal swing of P_CUFF_ and P_ES_ as the respective differences in pressure between the end-expiratory baseline and the maximal change during inspiration (Fig. 1), from 5 consecutive breaths without P_ES_ artifacts and patient-ventilator asynchrony at the end of each pressure support level.^[7]^

Throughout the entire pressure support titration procedure, which lasted approximately 30 minutes, the P_CUFF_ tracing was closely observed on the recorded waveform by the pressure transducer. The end-expiratory P_CUFF_ varied by less than 0.5 cmH_2_O across different pressure support levels, which was considered negligible. Therefore, no cuff volume manipulation was performed during the pressure support titration.

In our unit for mechanically ventilated patients, a light sedation is performed with a target Richmond Agitation Sedation Scale of -2 to + 1.^[8]^ In this case, 0.3 µg/kg/h fentanyl and 0.02 mg/kg/h midazolam were continuously infused via a central venous line. During the pressure support titration, the patient remained awake, calm, and was able to follow simple commands with the Richmond Agitation Sedation Scale of 0. During the procedure, the patient did not show signs of significant accessory muscle effort or respiratory distress.

As the pressure support decreased, the negative tidal swing of P_ES_ increased markedly, which indicated an increase in inspiratory effort (Table 1). Surprisingly, a similar trend of negative tidal swing was also found in P_CUFF_ tracings at pressure support levels below 11 cmH_2_O. At pressure support of 13 cmH_2_O and 15 cmH_2_O, the tidal swing of P_CUFF_ transitioned to a plain or slightly positive direction (Table 1). Linear regression analysis revealed a close correlation between the tidal swing of P_ES_ and P_CUFF_ (*R*² = 0.960).

**Table 1:** **Tidal swing of P**_**ES**_ **and P**_**CUFF**_**, as well as tidal volume, respiratory rate, and minute ventilation at different pressure support levels.**

Pressure support (cmH_2_O)	5	7	9	11	13	15
P_ES_ (cmH_2_O)	−12.7 (−15.7 to −11.5)	−12.6 (−13.5 to −11.9)	−11.0 (−11.6 to −9.6)	−8.5 (−9.4 to −7.4)	−7.0 (−7.3 to −6.8)	−4.8 (−5.4 to −4.7)
P_CUFF_ (cmH_2_O)	−1.2 (−1.3 to −1.0)	−1.0 (−1.1 to 1.0)	−0.5 (−0.6 to −0.5)	−0.2 (−0.2 to −0.1)	0.1 (−0.1 to 0.2)	0.2 (0.2 to 0.3)
Tidal volume (mL)	412 (365–430)	453 (403–468)	464 (447–488)	500 (487–530)	507 (503–517)	523 (512–537)
Respiratory rate (breaths/min)	22 (20–25)	23 (20–27)	21 (20–22)	18 (16–19)	16 (15–18)	17 (15–18)
Minute ventilation (L)	10.1 (8.6–10.7)	9.8 (9.2–11.7)	9.9 (9.2–10.2)	9.5 (7.9–9.7)	8.5 (7.9–9.4)	9.1 (7.9–9.5)

Data are shown as the median (interquartile range). A significant correlation was also found between tidal swing of P_ES_ and P_CUFF_ using linear regression analysis (*R*^2^ = 0.960).

PCUFF = endotracheal tube cuff pressure, PES = esophageal pressure.

During the downward adjustment of pressure support, tidal volume decreased, but respiratory rate increased, with a relatively stable minute ventilation (Table 1).

**Figure 2.**
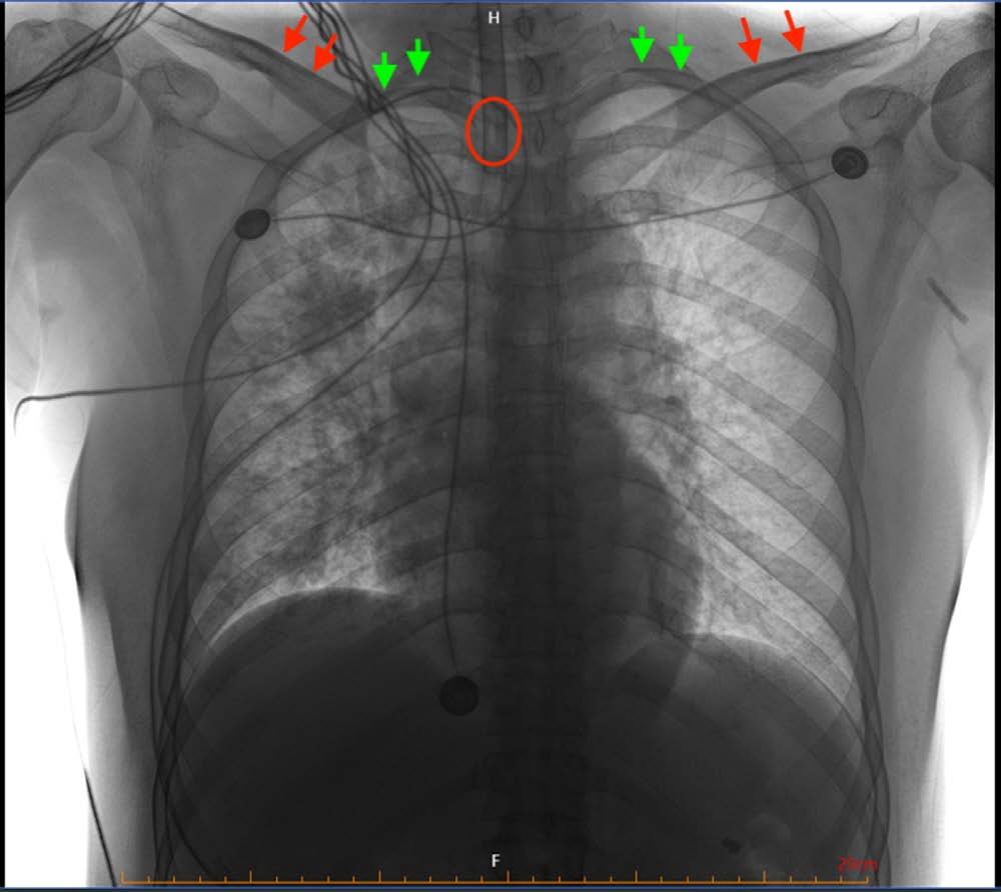
Chest radiography shows that the position of endotracheal tube cuff (red circle) is below the thoracic inlet, which are defined by the first rib (green arrows) and clavicle (red arrows).^[9,10]^

Chest radiography confirmed that the endotracheal tube cuff position was below the thoracic inlet and within the thoracic cage (Fig. 2).^[9,10]^

## 3. Discussion

P_ES_ measurements are used as the reference method for assessing inspiratory effort.^[3]^ Recently, P_AW_-based indices derived from airway occlusion maneuvers have been introduced to simplify the evaluation of effort without the need for additional equipment.^[4,5]^ The present case report suggests another potential method for effort measurement, namely the tidal swing of P_CUFF_ during the inspiratory phase under PSV.

In 1987, Badenhorst reported pronounced swings in P_CUFF_ below atmospheric pressure during spontaneous inspiration in patients undergoing continuous positive airway pressure.^[11]^ Although the author discussed that the negative swings of P_CUFF_ might reflect the decrease in pleural pressure induced by inspiratory effort, the phenomenon could not be confirmed because pleural pressure was not measured in the study.^[11]^ Thereafter, the studies relating to the endotracheal tube cuff primarily focused on the positioning of the cuff, pressure thresholds for seal, and the management of cuff leaks.^[12]^ In the present case report, P_ES_, and P_CUFF_ were simultaneously measured and recorded. Negative tidal swings of P_CUFF_, which resemble the tracings of P_ES_, were observed during the inspiratory phase at relatively low levels of pressure support (Fig. 1), and a significant correlation was found in the magnitude of pressure change between P_CUFF_ and P_ES_ across different pressure support levels (Table 1).

**Figure 3.**
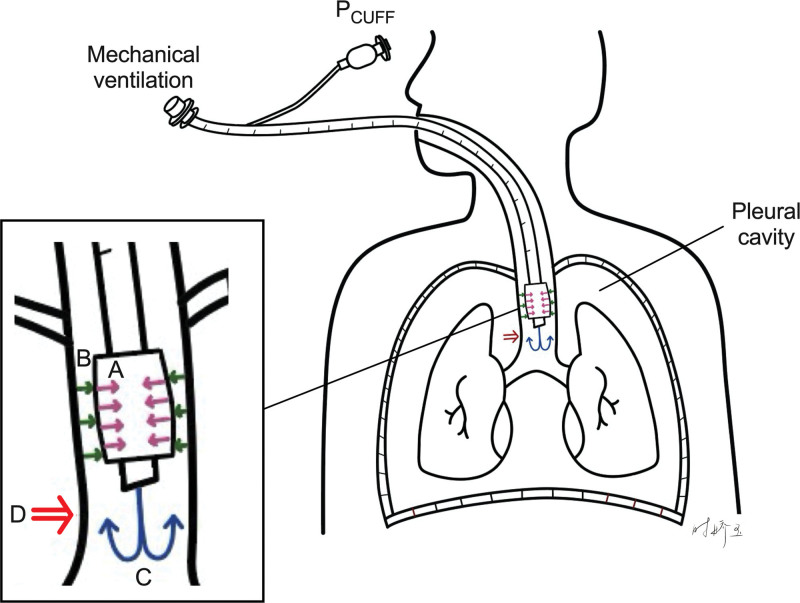
A schematic illustration of the influence factors on endotracheal tube cuff pressure (P_CUFF_) during pressure support ventilation, as long as the cuff is located within the thoracic cavity. (A) the elastic recoil pressure of the cuff induced by inflation (pink arrows); (B) the elastance recoil pressure generated by the tracheal wall (green arrows); (C) the airway pressure delivered by the mechanical ventilation (blue arrows); and (D) transmission of the pleural pressure around the trachea (red arrow). P_CUFF_ = endotracheal tube cuff pressure, P_ES_ = esophageal pressure, PSV = pressure support ventilation.

In the present case, we confirmed the intrathoracic location of the endotracheal tube cuff by chest radiography (Fig. 2).^[9,10]^ Theoretically, P_CUFF_ within the thoracic cage may be influenced by 4 sources of pressure: the elastic recoil pressure of the cuff induced by inflation; the elastance recoil pressure generated by the tracheal wall; the P_AW_ delivered by the mechanical ventilation; and transmission of the pleural pressure around the trachea as long as the intrathoracic position of the cuff (Fig. 3). During the short period of pressure support titration in this case, the cuff inflating pressure remained same, and the tracheal wall elastance might have been unlikely significant change. The increase in negative tidal swings P_ES_ during downward pressure support adjustment indicated an increase of inspiratory effort induced by decreasing ventilatory support (Table 1). Additionally, a close correlation was also found between tidal swings of P_CUFF_ and P_ES_. We speculated that the negative tidal swings of the P_CUFF_ might have been mainly due to a decrease in pleural pressure resulting from an increase of spontaneous inspiratory effort. However, whether the tidal swings of P_CUFF_ could be used as a surrogate for inspiratory effort assessment warrants further prospective diagnostic studies.

It is worth noting that the tidal swing of P_CUFF_ became plain or even positive at higher pressure support levels (i.e., 13–15 cmH_2_O in this case). This phenomenon may be attributed to the diminished inspiratory effort under high ventilatory support. Alternatively, the elevated inspiratory P_AW_ at high support levels may mechanically oppose negative cuff pressure deflections. These observations suggest an important limitation of the P_CUFF_-based assessment of inspiratory effort, namely, reduced reliability under conditions of high pressure support. Therefore, while P_CUFF_ might be promising as a noninvasive surrogate for monitoring inspiratory effort at moderate-to-low support levels, its application under high support settings requires further investigation.

## 4. Conclusion

Here, we report a case undergoing PSV demonstrating the relationship between tidal swings of P_CUFF_ and P_ES_. Given the clinical accessibility of P_CUFF_, we believe that this case report would advocate relevant clinical research on the use of P_CUFF_ for assessing inspiratory effort in patients under assisted mechanical ventilation.

## Acknowledgments

We want to express our special thanks to Ms. Jiao-Yu Shi, who permitted to be named, for her excellent work on drawing the Figure 3 for the manuscript.

## Author contributions

**Conceptualization:** Rui-Zhi Zhang, Ming-Yue Miao, Yun-Long Yue, Jian-Xin Zhou.

**Investigation:** Rui-Zhi Zhang, Shan-Shan Xu.

**Methodology:** Rui-Zhi Zhang, Shan-Shan Xu, Jian-Xin Zhou.

**Project administration:** Ming-Yue Miao.

**Supervision:** Jian-Xin Zhou.

**Visualization:** Rui-Zhi Zhang, Hai-Yang Liu, Jian-Xin Zhou.

**Writing – original draft:** Rui-Zhi Zhang.

**Writing – review & editing:** Rui-Zhi Zhang, Shan-Shan Xu, Yun-Long Yue, Jian-Xin Zhou.
